# Spreading and sustaining best practices for home care of older adults: a grounded theory study

**DOI:** 10.1186/s13012-014-0162-4

**Published:** 2014-11-07

**Authors:** Jenny Ploeg, Maureen Markle-Reid, Barbara Davies, Kathryn Higuchi, Wendy Gifford, Irmajean Bajnok, Heather McConnell, Jennifer Plenderleith, Sandra Foster, Sue Bookey-Bassett

**Affiliations:** School of Nursing, Faculty of Health Sciences, Aging, Community and Health Research Unit, Department of Health, Aging and Society, McMaster University, 1280 Main Street West, Room HSc3N25C, Hamilton, ON L8S 4K1 Canada; Aging, Chronic Disease and Health Promotion Interventions, School of Nursing, Aging, Community and Health Research Unit, Clinical Epidemiology and Biostatistics, McMaster University, Faculty of Health Sciences, 1280 Main St. W., Health Sciences Centre, Room 3N25B, Hamilton, ON L8S 4K1 Canada; Nursing Best Practice Research Centre, School of Nursing, Faculty of Health Sciences, University of Ottawa, 451 Smyth Road, Ottawa, ON K1H 8M5 Canada; School of Nursing, Faculty of Health Sciences, University of Ottawa, 451 Smyth Road, Ottawa, ON K1H 8M5 Canada; International Affairs and Best Practice Guidelines Programs, Registered Nurses Association of Ontario, 158 Pearl Street, Toronto, ON M5H 1L3 Canada; Aging, Community and Health Research Unit, School of Nursing, McMaster University, 1280 Main Street West, Hamilton, ON L8S 4K1 Canada

**Keywords:** Spread, Practice guidelines, Home care, Older adults, Grounded theory

## Abstract

**Background:**

Improving health care quality requires effective and timely spread of innovations that support evidence-based practices. However, there is limited rigorous research on the process of spread, factors influencing spread, and models of spread. It is particularly important to study spread within the home care sector given the aging of the population, expansion of home care services internationally, the high proportion of older adult users of home care services, and the vulnerability of this group who are frail and live with multiple chronic conditions. The purpose of this study was to understand how best practices related to older adults are spread within home care organizations.

**Methods:**

Four home care organizations in Ontario, Canada that had implemented best practices related to older adults (falls prevention, pain management, management of venous leg ulcers) participated. Using a qualitative grounded theory design, interviews were conducted with frontline providers, managers, and directors at baseline (*n* =44) and 1 year later (*n* =40). Open, axial, and selective coding and constant comparison analysis were used.

**Results:**

A model of the process of spread of best practices within home care organizations was developed. The phases of spread included (1) committing to change, (2) implementing on a small scale, (3) adapting locally, (4) spreading internally to multiple users and sites, and (5) disseminating externally. Factors that facilitated progression through these phases were (1) leading with passion and commitment, (2) sustaining strategies, and (3) seeing the benefits. Project leads, champions, managers, and steering committees played vital roles in leading the spread process. Strategies such as educating/coaching and evaluating and feedback were key to sustaining the change. Spread occurred within the home care context of high staff and manager turnover and time and resource constraints.

**Conclusions:**

Spread of best practices is optimized through the application of the phases of spread, allocation of resources to support spread, and implementing strategies for ongoing sustainability that address potential barriers. Further research will help to understand how best practices are spread externally to other organizations.

## Background

Improving health care quality requires effective and timely spread of innovations that support evidence-informed practices [1,2]. However, implementing and spreading best practices in health care organizations involves time-consuming, complex, and resource-intensive processes [3,4]. It can take years to implement and sustain innovations, and many attempts fail over time [1,5]. Further, there is limited rigorous research on the process of spread, factors influencing spread, and models of spread. Understanding how to rapidly and effectively spread innovations in and across complex organizations is vital to improving the quality of health care delivery and effectively using scarce resources [6,7]. This is the first study to develop a research-based model of the process of spread of best practices related to older adults within home care settings. In this paper, the literature on spread will be briefly summarized, the rationale for focusing on the context of home care will be described, and the results of a grounded theory study of the process of spread of best practices in home care will be explained.

While important advances have been made in implementation science, little attention has been paid to spread and scale-up of health innovations [8]. Although there are no widely agreed on definitions of the terms spread and scale-up, it has been suggested that scale-up is more commonly used in international health while spread is used more frequently to refer to improvement changes in high-income countries [1]. In this study, we use the definition of spread as “the process through which new working methods developed in one setting are adopted, perhaps with appropriate modifications, in other organizational contexts” [4] p. xxiii.

The concept of spread has received increased international attention in the past decade. In the UK, for example, the National Health Service has developed a change model that includes spread of innovation as a key concept, as well as a spread and adoption tool and a leader’s guide on sustainability and its relationship with spread and adoption [9]. In New Zealand, a position paper was developed to guide action on spreading health innovations [10]. In the US, a number of conferences and panels have been held to advance the science and practice of scale-up and spread of health programs, resulting in recommendations for practice, policy, and research [8], as well as key summary papers [1,2,11,12].

The study of spread processes has emerged from the literature on the diffusion of innovation in complex organizational settings [4,13-15]. Authors describe spread pathways as elusive and nonlinear with erratic, circular, or abrupt processes [16,17]. The limited literature on spread attempts to identify the approaches, strategies, methods, and models that characterize successful implementation of best practices and to account for how spread occurs internally within organizations and externally to other organizations or sectors [2,7,16,18].

There are a number of frameworks of spread [19,20]. One of these frameworks, developed through a literature review, series of discussions, and authors’ experience, is focused on community-based reproductive health service innovations in Asia, Africa, and South America [20]. The other framework was developed through a literature review and interviews conducted in organizations successful in spread [19]. This framework was used to spread operational changes to improve access for veterans to primary care and specialty outpatient clinics. No detail is provided related to the research methods used for conducting or analyzing interviews with spreading organizations. Some of the common concepts in these two spread frameworks include (a) the identification of a new practice, (b) leadership support, (c) organizational support, (d) communication of the innovation, (e) measurement and feedback, and (f) social, cultural, political, and economic contexts within which spread occurs. These frameworks are focused on health innovations in community settings, but not specifically in home care.

There is strong rationale for a focus on spread in home care settings. The proportion of older adults in the population is escalating worldwide and is associated with an increasing demand for and use of home care services [21,22]. Older adults, the largest users of home care, often have multiple chronic conditions including dementia and are particularly vulnerable to negative health impacts such as hospitalization [22,23]. Home care contributes to the quality of life and functional health status of individuals while also replacing expensive hospital care with client-preferred care in the home [24]. Finally, home care organizations experience unique challenges in care provision including wide geographical service areas, high turnover, and inadequate funding [22,23].

Many innovative services have been developed and evaluated to support older adults to live independently in the community [25,26]. Work in the UK has addressed key issues such as: supporting self management of older adults with chronic conditions and multimorbidity, ensuring quality and cost-effectiveness of care, healthy active aging, and a national care home research network [27-29]. While these examples provide valuable strategies and resources to support implementation of services to improve home care for older adults, they do not provide in-depth understandings of the process or factors associated with spreading these innovations between or beyond local sites or organizations [28].

The limited research that has been done on innovation spread in healthcare has primarily focused on acute care [30,31] or veterans outpatient clinics [19]. One case study examined the spread of two best practice tools in a US home health care organization and found that attributes of the tool determined the success of implementation and spread [32]. While the report provides valuable information about spread strategies, successes, and challenges, it provides little information about the research approach used and did not result in a theory or model related to spread of innovation in home care settings.

In Ontario, Canada, many home care organizations have been working with the Registered Nurses’ Association of Ontario (RNAO) to implement and disseminate best practice guidelines (BPGs) [33]. Some home care organizations are known as Best Practice Spotlight Organizations (BPSO®s), selected by the RNAO through a request for proposal process to implement and evaluate BPGs [34]. Researchers have studied this initiative focusing on evaluation of BPG implementation [6,35,36], leadership supports [37-39], client outcomes [40], and sustainability [41] but have not focused specifically on spread in home care settings.

In summary, there is limited research on the process of spread of best practices in home care settings. Qualitative research that results in a deep understanding of the process of spread in home care, including theoretical models of spread based on research data, has the potential to guide practice and policy that results in successful spread initiatives using scarce resources most effectively. The research questions in this study were as follows: What is the process used to spread best practices related to caring for older adults within home care agencies? What factors influence spread or non-spread?

## Methods

### Design

A qualitative rather than quantitative research design was ideal to address the research questions because this approach (a) facilitates exploration of little-known areas, (b) generates rich and detailed data that contribute to an in-depth understanding of an issue, (c) involves data collection in natural settings, (d) includes voices of participants themselves, and (e) through making the world visible has the potential to transform that world [42,43]. More specifically, a grounded theory qualitative approach is optimal to address questions of process and to move beyond qualitative description to the development of conceptual or theoretical explanations [42]. This theory development is “grounded” in data from participants who have themselves experienced the process. The resultant theory may help to explain practice or provide a framework to guide future research. The grounded theory approach of Strauss and Corbin [44] was selected over a number of other approaches [42] because it offers more clear guidelines for data analysis, is compatible with contemporary thinking reflecting a shift towards social constructivist ontology, and pays attention to contextual factors that influence the phenomenon being studied [45]. This was the ideal approach to develop an understanding of how best practices related to caring for older adults are spread within home care agencies and the factors that influence spread.

### Sampling and recruitment

Purposive sampling was used to determine information-rich data sources (settings and participants) for participation in the study [44]. Consistent with grounded theory methods, theoretical sampling was used. Theoretical sampling is described as data gathering that is driven by concepts identified from the evolving theory and involves going to people, places, or events to more fully understand something that is only partially known [44]. Sampling continued until data saturation was reached.

#### Settings

Four home care organizations in Ontario, Canada, that had implemented best practice guidelines related to caring for older adults were purposively sampled (see Table 1). The organizations were included if they had spread, were in the process of spreading, or planned to spread tools and processes related to these best practices within their organization. The organizations represented diversity in their size, type of home care providers, and practice guideline (pain, venous leg ulcers, falls prevention). Three organizations provided home care services directly in clients’ homes, while one organization coordinated the provision of home care services.

**Table 1:** **Description of home care settings (** ***n*** **=4)**

**Site**	**Type of organization**	**Clients served/year**	**Employees**	**Year RNAO BPSO designate** ^**a**^ **granted**	**Topic of guideline implemented**
1	Home care provider (for-profit)	65,000–75,000	RNs, RPNs, PSWs	2012	Pain
2	Home Care Coordinator (CCAC)^b^ (government funded, not-for-profit)	65,000–75,000	Case managers (RNs, rehabilitation therapists and assistants)	2012	Venous leg ulcer
3	Home care provider (not-for-profit)	100,000–125,000	RNs, RPNs, PSWs, rehabilitation therapists	2006	Falls prevention
4	Home care provider (not-for profit)	100,000–125,000	RNs, RPNs	2006	Falls prevention

^a^Best Practice Spotlight Organizations (BPSOs) are health care and academic organizations selected by the RNAO through a request for proposal process to implement and evaluate the RNAO’s BPGs. Successful organizations begin with a 3-year candidacy period in which they implement and evaluate BPGs after which they become designate organizations. The initiative now has designate organizations in a number of countries worldwide.

^b^CCACs use case management to arrange access to home care providers for all in-home services including professional and home support services. Within limited budgets set by the provincial government, CCACs purchase home care services from local non-profit and private-for-profit community-based agencies that compete for service contracts through a competitive process.

#### Participants

Within each home care organization, we purposively sampled diverse participants from all levels of the organization (directors/managers, practice guideline leaders, champions, and frontline service providers). We expected a sample size of 8–10 individuals per site, interviewed twice (64–80 interviews), to be adequate for data saturation. This estimate was based on published grounded theory studies in health care that included three sites each and completed 44–120 interviews [46-48]. Consistent with theoretical sampling approaches, as the analytic process continued and the theory was developed, we returned to the field to interview more frontline providers to better understand their experiences. Interviews were an average of 1 h in length. A total of 84 interviews were conducted with 46 participants: 44 interviews were conducted at time one and 40 at time two.

#### Recruitment

The researchers worked closely with trained recruiters from the four organizations to identify potential participants who were given an information letter and invited to participate on a voluntary basis. Upon acceptance, recruiters sent participants the consent form to review.

### Data collection

Two semi-structured audio-taped interviews were conducted with each participant approximately 1 year apart (i.e., February-May 2012, January-April 2013). Interviews were conducted by the Principal Investigator (JP) and/or the Research Coordinators (JPle, SBB).

The development of the semi-structured interview guide used in the first interview was based on a review of the literature and experience of the team with previous BPG research. Participants were asked to describe their experiences with and perceptions of the process of spread (interview guide available on request). Interview questions addressed topics such as the rationale for selecting the particular guideline, tool development, early implementation of the innovation, participant role in the spread process, the process of spread, and facilitators and barriers to spread. In the second interview, participants were asked to discuss their experiences with and perceptions of the spread process in the intervening year since the first interview. Preliminary analysis of the data from the first set of interviews was used to develop lists of antecedents, spread strategies and processes, benefits, and facilitators and barriers to spread (4–17 items in each list, available on request). Participants were asked to review these lists and identify the items most important for their own spread process and why.

### Data analysis

Data from all interviews were transcribed verbatim, cleaned, and analyzed using NVivo 10 software. Data were collected and analyzed concurrently using strategies consistent with a grounded theory approach [44]. Open coding was used to identify major concepts and categories of information, as well as properties and dimensions of categories. Each transcript was reviewed and analyzed for similarities and differences using a line-by-line approach. A codebook was developed with a list of codes, code definitions, and sample quotes to represent each code. The codebook was refined throughout the analysis process. Axial coding was used to identify the phases of spread and core categories of facilitators of spread [44]. Sub-categories were created around the core categories. A flow chart representing the internal and external spread activities and processes for each organization was developed. Selective coding was used to integrate and refine categories to form the grounded theory and the visual model of the theory. Constant comparison of data across participants and organizations was completed to enable identification of similarities and variations in the patterns found in the data [44]. Numerous versions of the model were developed over time, discussed and refined with the research team, and compared with the original data.

Detailed memos of thoughts and perceptions of the data as they were collected and analyzed were maintained. The research team maintained written records of analytic ideas, concepts, and decisions made throughout data analysis.

Study rigor was also ensured through the use of member checking. In December 2013, the researchers followed-up with participants from each organization (ensuring representation of different categories of staff) to discuss the organization-specific flow charts of the spread process and to discuss the draft model of spread. Participants provided valuable feedback and suggestions for change. Overall, participants indicated that the model represented the spread process in their organization. One participant said ‘This feels familiar to me. It is exciting to see something put into the model that reflects what we did’.

Transferability of study findings was enhanced by the multi-site design and variety of best practices that were studied. Truth value or credibility was enriched through triangulating across data sources and data collection procedures, member checking, and deliberately trying to discount or disprove conclusions about the data (i.e., negative case analysis of the one organization where there was minimal spread). Collecting data from participants at two points in time assisted with data source triangulation, whereas having research team members review main categories and the model to confirm interpretation assisted with investigator triangulation.

### Ethical considerations

Ethics approval was obtained from McMaster University Hamilton Health Sciences Research Ethics Board on December 14, 2011 (project number 11-555) and from each participating site. Research team members ensured informed consent, collection of signed consent forms, and protection of participant confidentiality. Each organization received a small amount of funding to support their assistance with recruitment.

## Results and discussion

### Participants

Participants included 46 individuals from the four organizations, all but one of whom were female (see Table 2). Participants were predominantly over the age of 40 (80%), had a nursing education (72%), and worked full time (80%). Participants held a variety of positions within the organization: frontline (41%), management staff (26%), resources staff (17%), and senior management (15%). The mean length of time worked at their current organization was 9 years.

**Table 2:** **Demographic characteristics of participants (** ***n*** **=46)**

**Variable**	**Number (%)**
Gender	
Female	45 (97.8)
Male	1 (2.2)
Age (years)	
≤40	9 (19.6)
41–50	17 (37.0)
51–60	18 (39.1)
≥61	2 (4.3)
Position	
Frontline (RN, RPN, case managers)	19 (41.3)
Management staff (managers)	12 (26.1)
Resources staff (educators, clinical resource nurses, advanced practice nurses)	8 (17.4)
Senior management (directors)	7 (15.2)
Employment status	
Full-time	37 (80.4)
Part-time	9 (19.6)
Education	
Diploma in nursing	15 (32.6)
Bachelor’s degree in nursing	13 (28.3)
Master’s degree in nursing	5 (10.9)
Other bachelor’s degrees	3 (6.5)
Diploma plus other education	6 (13.0)
Other master’s degrees	4 (8.7)
Mean length of time at current position (years)	6.13
Mean length of time at current organization (years)	8.82

### The model

**Figure 1:**
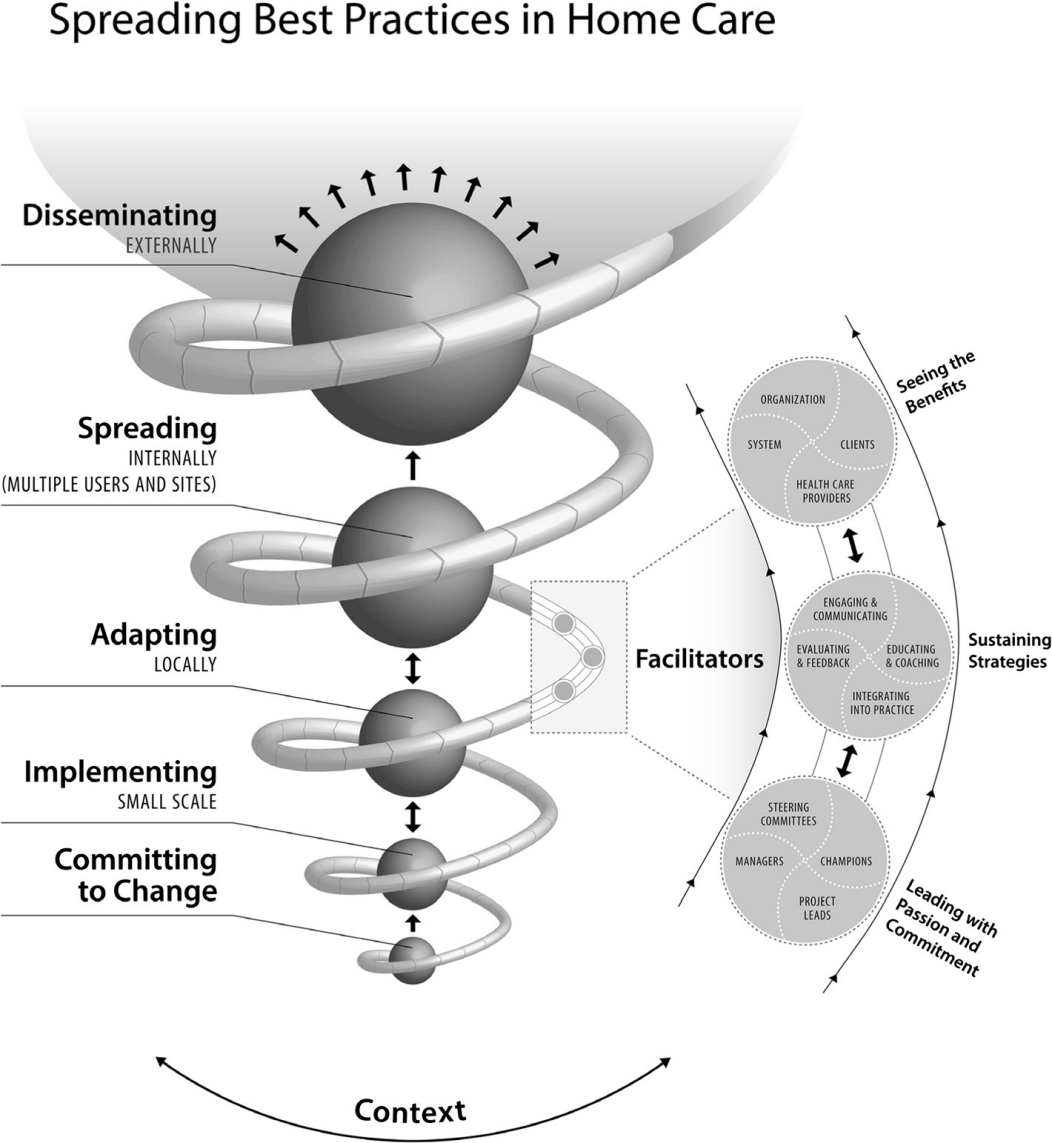
**Model of spread.**

A grounded theory of the spread process was developed, and the corresponding model is illustrated in Figure 1. The theory includes a five-phased process of spread, three key factors that facilitated this process, and contextual factors within which the process occurred. The five-phase process included (a) committing to change, (b) implementing on a small scale, (c) adapting locally, (d) spreading internally, and (e) disseminating externally. The three key factors that facilitated the spread process included (a) leading with passion and commitment, (b) sustaining strategies, and (c) seeing the benefits. The spread process occurred within a community care context characterized by wide geographic distribution of staff, heavy workloads, high turnover, and constant change. In-depth data analysis revealed that three of the four organizations demonstrated internal spread (see Table 3 for detailed descriptions of the spread process for sites 1 and 2). Data analysis for the fourth organization indicated that there had been minimal spread of the innovation (see Table 4 for a description of the non-spread site). In the following sections, each of the phases, facilitating factors and contextual factors are described and illustrated with quotes identified by organization and participant number (e.g., participant 01-03 is the third participant from organization 01).

**Table 3:** **Description of spread process sites 1 and 2**

**Site 1**	**Site 2**
Background	Background
This accredited for-profit agency provides home care services in urban, rural, and remote communities throughout Ontario, Canada. There are ten geographically dispersed branch offices. The team includes nurses, rehabilitation therapists, and community support workers who deliver a broad range of services including chronic illness management, nutrition, physiotherapy, personal grooming and support, palliative care, and relief for caregivers.	This accredited agency is a Community Care Access Centre (CCAC), 1 of 14 operating in Ontario, and funded by the Ministry of Health and Long-Term Care. CCACs provide a first point of contact for public access to government-funded home care, community services, and long-term care homes. This organization has five geographically dispersed branches. The CCAC’s care coordinators provide coordination services in home and hospital settings and include services related to older adults, palliative care, pediatric care, rural health care, and information and referral. CCACs provide funding to community agencies that deliver nursing, rehabilitation, and other services in the home.
Committing to change	Committing to change
Site 1 became a Registered Nurses’ Association of Ontario Best Practice Spotlight Organization in 2009, some years before the start of the pain spread initiative. This involved a *commitment* to implement and evaluate four best practice guidelines over a 3-year period. The *project lead*, who was a manager, received a fellowship that enabled her to focus on developing leadership and skills related to chronic pain best practices. Her *passion* about this topic and guidelines in general led to discussions with senior leadership and a decision to pursue the implementation and spread of the pain assessment and management guideline. A *steering committee* composed of frontline staff, *champions*, managers, and the project lead was established to lead the process of developing and implementing a tool related to pain. Using the RNAO guideline on assessment and management of pain as a basis, a tool was developed to support the assessment and management of pain for home care clients. The three-page tool included a detailed assessment of pain characteristics and assessment findings, client goals, and resources. The tool also included a pain assessment and management flow sheet that addressed use of medications and alternative pain management strategies, client reports of impact of the intervention, and side effects of treatment. The steering committee held *workshops* with frontline staff and champions to present the tool and obtain feedback prior to its implementation. This preliminary *feedback* was used to make some minor revisions to the tool.	Site 2 became a Registered Nurses’ Association of Ontario Best Practice Spotlight Organization in 2009. This involved a *commitment* to implement and evaluate best practice guidelines over a 3-year period.
	A *steering committee* composed of care coordinators, a service manager, and a clinical expert was established to lead the process of developing and implementing a case management decision process for venous leg ulcers. Using the RNAO guideline on Assessment and Management of Venous Leg Ulcers, tools were developed to support the care coordination of service providers (nurses and others) who were providing care to clients with venous leg ulcers. These tools included a detailed care pathway to guide service provision in light of degree of leg ulcer healing, posters related to the wound care pathway, and laminated flip cards with detailed guidelines for implementing the new care pathway. An advanced practice consultant was hired to provide clinical expertise related to wound care and assist with staff education.
Implementing on a small scale	Implementing on a small scale
Site 1 implemented the first version of the pain assessment and management tool in two of its ten branches, one in an urban location and one in a more rural location. The project lead and Clinical Nurse Educators (champions) conducted *in-services* for all nursing staff at these locations to learn about the pain tool. Weekly emails were sent to the staff that included reminders about the tool and clinical vignettes that gave actual examples of how the tool was implemented and *positive impacts* in selected client situations. The project lead provided informal one-to-one education for some frontline staff, both in the office and at joint home visits. At monthly team meetings, the project lead, managers and champions had informal discussions with the staff about the use of the tool.	Site 2 implemented the first version of the venous leg ulcer tools in one branch in an urban area. The project lead and steering committee members conducted *train-the-trainer sessions* for *champions* related to this new tool. The project lead and other senior level staff provided informal one-on-one *education sessions* about the new tool with care coordinators.
Adapting locally	Adapting locally
Formal and informal *feedback* about the tool was obtained from frontline staff and discussed at steering committee meetings throughout the early implementation phase. The project lead conducted some client visits on her own and identified challenges in using the tool in the actual client homes. In particular, the tool was lengthy for both clients and nurses to complete, and some clients expressed concern about the burden of completing this assessment. This ongoing feedback resulted in the steering committee and project lead making *revisions to the tool* including shortening it, simplifying the assessment and response items, and using check boxes instead of open-ended responses in other spread sites.	Chart audits were completed by the Advance Practice Consultant during the early implementation, and results were shared with care coordinators.
The project lead also revised her educational strategies from early implementation sites to later ones, moving from more didactic sessions to discussions of real client scenarios in small groups. She encouraged nurses to describe results of using the pain tool, problem solve possible interventions together and then followed up on the results of those interventions. The project lead took on major responsibility for education and communication about the pain tool while implementation occurred at the first two sites. She regularly visited the sites, met with managers, nurses and champions, conducted joint visits with nurses and coached them on the use of the tool. However, in consultation with the steering committee and leaders, she recognized a need to increase the participation and responsibility of *local nurse managers* at the other eight sites to better facilitate spread. Thus, education and support was provided to local managers to better enable them to support the spread of the pain tool in their own branches. The project lead then followed up with managers regularly to discuss results of chart audits and other indicators of spread.	
Spreading internally	Spreading internally
Over the next 6 months, the tool was spread to the other eight geographically dispersed branches. The tool was *integrated into all client charts* on new admission. The project lead and managers at the local branches provided in-service education for all the staff related to the tool. The staff were paid to attend these educational sessions. The local nursing managers dedicated time during at least two of the monthly team meetings to discuss the pain tool and engage the staff in discussions of their experiences and *suggestions for change*. Several newsletters included articles written about the tool by the project lead. The tool was often mentioned in weekly emails to the staff. A pain guideline package was created and posted on the portal website for the staff. Nurse managers had *coaching sessions* related to the tool with the staff in the office and in client homes. The tool was *integrated* into the employee orientation plan as well as the annual performance appraisal process. Components of the tool were *integrated* into the palliative and oncology care plans. *Chart audits* that included the pain assessment tool were completed by steering committee members and frontline staff. Results of these audits, such as extent of use of the tool, were shared with managers so they could share these at team meetings and address gaps in care with frontline staff. Stories were widely shared about the *benefits of using the tool*, such as actual changes in client pain and quality of life, and improved responses of family physicians when nurses shared the results of their pain assessments and ideas for managing client pain.	Over a 2-month period, the new tools were spread to four geographically dispersed sites.
	Steering committee members held question and answer sessions with staff members to obtain feedback and subsequently streamlined the case management pathway to make it simpler and easier to use. Leadership developed and monitored some key performance indicators through an electronic database and shared results with frontline staff at staff meetings.
Disseminating externally	Disseminating externally
The pain tool and processes of spread were *shared* with a number of external agencies and at a number of events. For example, the tool was shared with the home care coordinator agency that contracted site 1 to provide care. The project lead and a manager presented the pain tool at an Ontario Palliative Care conference as well as at RNAO workshops and teleconferences. The project lead and one of the managers integrated content about the tool into a Comprehensive Advanced Palliative Care Education course offered in the community through hospice care consultation teams.	The venous leg ulcer care pathway and processes of spread were shared with a number of external agencies and at some events. For example, the project lead met one-on-one with local family physicians and surgeons to describe the new pathway. Care coordinators working in hospital settings to facilitate the discharge process to home care shared the tool with the hospital-based Skin and Wound committee. The innovation was also shared with a regional best practice group including public health and hospital staff and at a variety of workshops such as the Ontario Association of Community Care Access Centre conference. A paper was published describing the new pathway and the processes used to implement and spread this innovation. Finally, the project lead was consulting with the Ontario Association of Community Care Access Centres, a provincial group representing all 14 CCACs, on the development of a consolidated wound care pathway that could be used across all CCACs.

**Table 4:** **Description of non-spread site**

**Categories**	**Description**
Barriers to spread	The findings of the non-spread site confirmed the importance of the facilitators identified in the spread model, largely by their absence. Participants at the non-spread site stated that the organization was committed to falls prevention strategies and initiatives. However, they were *not actively implementing and spreading* the specific falls prevention tool under study. Frontline staff recalled having a presentation related to the tool at a staff meeting, but they commonly stated they were not using the tool: “I haven’t actually used it…it’s kind of obvious that a lot of us are not using it” (04-01). They described that they were *not clear* if the tool was to be used with all clients or only with selected clients. Common responses to questions about the development, piloting, and spread of the tool included: “I couldn’t tell you, I don’t know” (04-02).
	One of the key barriers to spread at this site was identified as the lack of a *project lead, steering committee*, *and champions* related to the initiative. At this site, the project leader left the organization shortly after the introduction of the falls tool and was not replaced, leaving a critical *gap in leadership*: “We’re in a transition time right now because we just lost our clinical educator…so I know that she was working on it first…and I’m not sure where that is going right now… because I don’t think there is anyone else involved in that” (04-01). When participants were asked if they were aware of any working group or champions to assist with the roll out of the tool, they responded with: “No, not that I’m aware of” (04-06). Participants were also *uncertain about the benefits* of using this tool, or any audit and feedback mechanisms related to the use of the tool: “I couldn’t answer that…I would assume they are checking these things in the chart audits…I don’t know” (04-04).
Barriers to sustainability	At the second interview, participants referred to issues that prevented sustained emphasis on the new tool, including manager turnover, new priorities, and lack of ongoing education: “We’ve had a lot of changeover in our management so there’s other priorities…We haven’t really had any education on it since a year and a half ago” (04-01).
Advice for future spread processes	The advice of frontline staff in relation to future spread projects directly addressed many of the limitations experienced in their own spread process: “It goes back to resources, you have to have *champions*…have your *core group* that figure out what’s in it for them, how it can *make their life better or their client’s life better*…you need the *leadership* resources to get that going” (04-09). Participants also identified the need for *involvement of frontline staff* in the change process, information about the benefits of using the tool, and ongoing *feedback*: ‘Bring in the staff and ask for their feedback and also present the whole picture to them. Don’t just say ‘here’s a guideline we’ve been told to implement’, give them some information: ‘We have found that this guideline has led to this much decrease in falls, it hasn’t increased workers hours by any length of time, it’s actually…made their day more efficient or clients are happier’…Give them some real feedback on how things have worked. And for sure I would ask them first how do you feel about it?’ (04-04).

### Phases of the process of spread

The phases of spread reflected a number of process characteristics including sequences, cycles, and spirals [49] (see Table 5 for a description of sequences, cycles, and spirals). The innovation that was spread included both tools related to guideline recommendations as well as processes to support spread, hereafter referred to as ‘the innovation’.

**Table 5:** **Examples of sequences, cycles, and spirals in the spread process**

	**Examples**
Sequences	In all three spread sites, participants described sequences of the spread process, where certain phases or activities occurred in a sequential order. This is illustrated in the model with the sequential movement from committing to change through to small-scale implementation, adapting locally, spreading internally to more sites and users, and finally disseminating externally.
Cycles	In all three spread sites, participants described cycles of activities, in particular educating the staff about the tool, having the staff try the tool in practice, receiving their feedback about the tool, using that feedback to revise the tool, and then having the staff try out the revised version, and going through the process again. This is illustrated in the model with two-way arrows between the phases of implementing on a small scale and adapting locally, and adapting locally and spreading internally.
Spirals	Participants at the spread sites described ways in which the spread activities gained momentum or accelerated/spiraled over time. This is illustrated in the model with the increasing size of sequential phases (circles) and the increasing size of the spiral rope over the spread phases. Participants explained that the process of implementing on a small scale took longer than later internal spread activities, as this involved more revisions to the tools, and testing of different approaches. At site 1, there was momentum as the tool was incorporated into other practice areas reaching a much larger client population: “We really learned that we could incorporate the pain assessment or management…into other flow sheets, our palliative care flow sheet, our oncology wound, so on. We’ve incorporated pain management more into our wound program which spans 60% of our clients…so it has definitely spiraled into a much bigger population than just…pain management.”

### Committing to change

The first phase of the spread process, committing to change, represented organizational commitment to the change through: (a) commitment to evidence-informed practice, (b) previous lessons learned with guideline implementation, and (c) recognition of practice needs and gaps. All four organizations had an explicit commitment to evidence-informed practice, with a particular focus on implementing, evaluating, and spreading best practice guidelines. Each had gone through the process of becoming a RNAO BPSO® designate and had developed and carried out specific plans for implementing a number of best practice guidelines over 3 years and committed to spreading and sustaining those practices. The commitment to change also involved allocating resources such as funding for a dedicated project leader and targeted educational activities. Each organization drew on previous lessons learned from guideline implementation as a foundation for the current spread process. Participants described both successful and unsuccessful experiences with guideline spread and recognized the value of initially trying the innovation on a smaller rather than larger scale.

A commitment to change also involved the identification of practice needs and gaps specific to the guideline topic. As these needs and gaps were recognized broadly within the organization, there was a readiness to take action. In one organization, there was a recognized need for improved consistency of care across geographic boundaries.

There was a lot of disparity among supply usage, healing times, length of stay on nursing services, delays in wound healing. So that promoted the need to have some consistency and be fiscally responsible and have that equitable access to services in such a large organization. (participant 02-11)

### Implementing on a small scale

The second phase of the spread process, implementing on a small scale, involved trying out the innovation in selected sites or branches prior to spreading the change throughout the larger organization. The three spread organizations had a steering committee, dedicated project leads, champions, and managers who were very involved in planning and implementing the innovation. These individuals and groups reviewed the best practice guideline, identified key practice recommendations to focus on, and developed practice tools based on these recommendations.

The complexity of tools developed varied by organization: (a) 14 items on home assessment to prevent falls, (b) 9-item form related to falls risks in and outside of the home, (c) a more complex pain flow sheet that included an assessment of pain characteristics and descriptors, contributing factors, impact of pain, client perceptions of pain and pain goals, resources, interventions taken, and evaluation of their impact, and (d) venous leg ulcer care pathway and related decision tools for case managers.

A decision was made, usually by senior managers in consultation with project leads and managers, to try the tools in selected sites within each organization. Steering committees, project leads, champions, and managers used communication and education strategies with frontline providers to encourage use of the new tools. Participants explained that this phase involved a cyclical process of trying the best practice tools and processes, getting feedback and adapting to local contexts, and then retrying. Implementing on a small scale was viewed as creating a ‘solid base that was proven to be effective prior to the rollout to the larger organization’ (participant 02-03). This phase was viewed as saving time and resources by ensuring the innovation would be accepted more broadly and that all stakeholders had opportunity for input.

You could waste a lot of resources and time trying to roll something out across the board… So I would definitely say a pilot site, get some feedback, get people’s ideas…because then the nurses feel like they are getting a chance to have some input and offer some advice. (participant 04-04)

### Adapting locally

The third phase of the spread process, adapting locally, involved the careful review of feedback on the successes and limitations of the small-scale implementation and revising both the tool and the processes used to spread to local contexts. Participants described strategies used to collect feedback from frontline users including formal written evaluations, team meetings, and informal discussions with managers and project leads. Participants emphasized the importance of obtaining feedback from the frontline providers, nurses, and others who used the best practices with clients to determine the feasibility of the practice change and understand the revisions that were needed.

You want to make sure what you are doing is doable and I find that frontline staff will be brutally honest and let you know if something is not doable and why. And, if that happens, you want them to be part of the process in trying to figure out how to get that done. (participant 03-11)

Project leads, managers, and steering committees reflected on the feedback and adapted or tailored the innovation to better fit the specific contexts of the sites. Participants used terms such as ‘work out the flaws’, ‘fine tuning’, and ‘work out the kinks’ as they adapted the innovation to improve staff buy-in and spread to other areas. Adaptations made included shortening tools, simplifying scoring methods, and ensuring there was a good fit with what providers were already doing.

Participants explained that ‘different areas had different strategies’ (participant 01-10) to spread best practices. There was diversity in the individuals who supported the spread process at different sites and the educational approaches used to support spread based on resources available and accepted practices. Communication strategies to support spread also varied with some sites using, for example, weekly electronic updates and newsletters, and others using team meetings.

### Spreading internally

The next phase of spread, spreading internally, involved moving the innovation throughout the organization to all sites and users. This process varied, with one setting moving the innovation to all additional sites at approximately the same time, whereas two sites used a phased approach to internal spread.

We get a calendar…it’s going to be rolled out in [Location A] here and it’s going to [Location B] this date and will be down in [Location C] that date. Because our advance practice team is a small team and they have to be available to go to many, many places across the province…it would be weeks or sometimes even months. It also allows each site to get their staff in place, to bring them in for education, and bring two hundred people together in the same room. (participant 03-07)

Participants talked about the value of engaging spread sites early by sharing information about the innovation well before the internal spread phase. Leaders from the early implementation site provided education to representatives (e.g., managers, champions) from spread sites on ways to use the tool and strategies to support its use by frontline staff. These representatives then used multiple strategies to educate frontline staff at their sites. The new tool was placed in all client admission charts or made readily available to users. Participants emphasized how important it was to ensure manager commitment to change in this phase.

### Disseminating externally

While three of the four organizations demonstrated internal spread, none of the organizations demonstrated external spread where the innovation was adopted by external organizations. However, three organizations demonstrated external dissemination defined as dissemination of knowledge that is focused primarily on communicating research results by targeting and tailoring the message to particular audiences and which precedes implementation or application of that knowledge [50]. Participants described sharing of spread experiences through presentations at workshops and conferences as well as through published articles. One of the participants described the plans for a new province-wide project to consolidate wound care pathways for all home care agencies and the potential for their organizational wound care work to inform the development of that initiative.

### Factors facilitating the spread process

**Figure 2:**
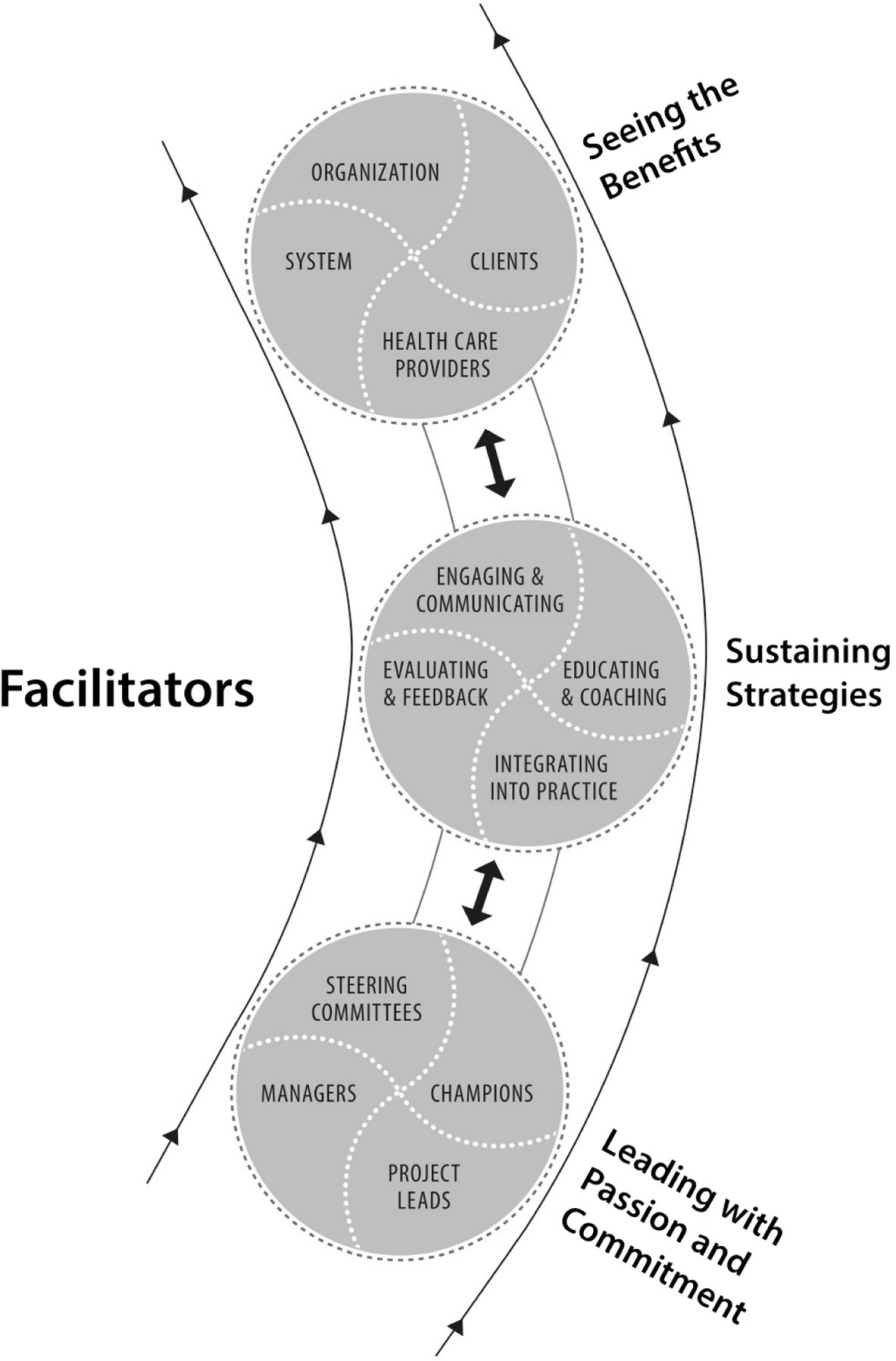
**Facilitators of spread (close-up from model of spread in Figure** 1 **).**

Three factors facilitated the spread process: (a) leading with passion and commitment, (b) sustaining strategies, and (c) seeing the benefits (see Figure 2). These factors were clearly evident in the three organizations where internal spread occurred, but were largely absent in the non-spread site (see Table 4).

### Leading with passion and commitment

Leading with passion and commitment refers to the leadership approach of project leads, champions, managers, and steering committees. Some individuals held more than one of these roles concurrently. Project leads were managers or resource staff who led the spread process with passion and commitment; their leadership was seen as a driving force that created a necessary following for successful spread:

She was persistent and when you have a vision you share it with other people and you’re passionate about something, they tend to feel your passion, too, so that that was probably the biggest driving force that contributed to her success with it…she just created all these followers. (participant 01-10)

Participants explained that the success of project leads in facilitating spread was associated with their ability to develop trusting and respectful relationships with frontline providers. Project leads played roles such as setting timelines for the spread process and ensuring accountability to goals. Participants emphasized that project leads required release time for their roles, supported by organizational or other funding.

Champions were identified as individuals at all levels of the organization (e.g., frontline staff, managers, administrators) and in all sites or locations who were passionate about the innovation and acted as resource or ‘go-to’ persons.

Champions for sure, the passion and the energy and the respect, and the resource within the organization. Every organization needs a person on a day-to-day basis that they can go to because as they are learning new information, they need that reassurance. (participant 02-01)

Frontline champions were seen as particularly vital in influencing their peers to adopt the innovation as it spread throughout the organization. Their sense of ownership and willingness to help their peers ‘work through a [client] issue’ (participant 02-11) were important to get grass roots buy-in.

Managers were members of the steering committee and played facilitating roles as educators, mentors, and role models for frontline staff. They provided both formal and informal education related to the innovation. Frontline providers described how valuable it was when managers would go with them on home visits, interpret the best practice tool, and walk through its application.

My nurse manager came out on a couple of home visits and she said ‘this is the new pain sheet’ and she just went through every page with me and ‘this is how it works and this is how to use it.’ So that was the most effective thing for me because otherwise it was just this sort of vague, you pull it out of the chart and it looks so daunting and time consuming. (participant 01-06)

Steering committees were usually composed of frontline providers, managers, project leads, champions, and clinical resource staff. In the early phases, steering committees carefully reviewed the BPG, identified relevant recommendations, and developed tools to be implemented. They ensured the development of a ‘strong communication plan’ and the sharing of a ‘unified message’ (participant 02-11) about the practice change. In later phases of spread, the steering committee planned the small-scale implementation, obtained and reviewed feedback, and guided the adaptation of the innovation for internal spread. Participants explained the value of diverse perspectives in moving the spread process forward.

Having a steering committee really made a big difference because we were all from different areas…and we all have different connections…working as a team and pulling all of this together from everybody’s different perspectives and experiences and input really made a big difference. (participant 01-10)

### Sustaining strategies

Interviews conducted a year after the initial interviews highlighted the importance of sustaining strategies for successful spread. Participants gave examples of diminishing focus on the innovation following the spread process, including less funding for the project lead, frequency of steering committee meetings, time committed by champions and managers to the innovation, and educational time. Four strategies used throughout the spread process not only facilitated the spread process, but also helped to sustain the continued use of the innovation once spread had occurred: (a) engaging and communicating, (b) educating and coaching, (c) integrating into practice, and (d) evaluating and feedback.

Engaging and communicating strategies were used by project leads, managers, and champions to engage frontline providers in discussions of the innovation. They discussed what worked and what did not, sought out positive examples of practice change and recognized and reinforced those changes.

All participants described diverse educational strategies to support spread that were used by project leads, champions, managers, steering committees, and resource staff. These strategies included team meetings, orientation sessions, one-on-one sessions, electronic media, newsletters, mentoring, and coaching. One-on-one mentoring and coaching by managers, champions, and project leads, often in client homes, was viewed as one of the most effective change strategies.

The more we’ve learned that the mentoring and coaching is really…the part that’s effective. That’s the part that makes a difference in changing the practice of nurses. (participant 01-01)

The third sustaining strategy, integrating into practice, involved incorporating the innovation into documentation, care paths, policies, and procedures so that it was ‘woven into the very fabric of what we do’ (participant 01-04). Participants indicated that the tools had to be ‘right there’ and ‘in front’ of providers who were busy and often overwhelmed with paperwork. Further, the tools had to be structured to clearly guide practice.

The spread process was also facilitated by the sustaining strategy of evaluating and feedback. Participants emphasized the importance of listening to feedback from frontline providers and ensuring their voices were heard and acted on in the change process. However, participants noted that most clients’ charts were hard copies that remained in the home until discharge, making it challenging to monitor both the degree of spread of the innovation and the client outcomes related to spreading the innovation.

The most important barriers…feedback would be the big one…I don’t really know that the loop closes very well. It would be nice to have that feedback and to hear that it’s making a difference. (participant 03-08)

### Seeing the benefits

The third facilitating factor of the spread process was that participants were seeing the benefits of the change at multiple levels of impact, namely the client, health care provider, organization, and system. While it was challenging to obtain impact information, seeing these benefits created an ongoing momentum for the spread process.

What makes this one easy to spread is the fact that very quickly the nurses realize how much they can improve the disease experience for their patients. So they really help buy-in because they feel empowered to make a difference. And pain is such a distressing symptom that to walk away from that visit and know you have made a difference is really a powerful thing. (participant 01-04)

It was important for providers to see the benefits of the change for their own practice and recognize that the innovation was not adding to heavy workloads but making their practice more effective. At the organizational level, there was a recognition that the practice change resulted in a more consistent approach to client care and helped to ensure equity in access to care. Finally, seeing the benefits of change at a system level facilitated the spread process:

Ultimately it’s all about the client....the ultimate benefit is that we are preventing clients from injuries and preventing readmission to hospital and all those things impact the health system as a whole. (participant 02-11)

### Contextual factors

The context of home care practice was riddled with barriers to the spread process. These barriers operated at individual, organizational, and system levels and were addressed, in part, through the effectiveness of the facilitating factors previously described. At an individual level, barriers included resistance of the staff to integrate new practices in the face of already heavy workloads and documentation requirements. Some of the organizational barriers included staff and manager turnover, constant change, and the lack of electronic records.

Participants explained that the context was rife with ongoing new initiatives mandated by employers and funders. The nature and frequency of these changes detracted from the necessary focus on the spread process and ongoing sustainability of the innovation. Further, most settings did not have electronic health records which made it hard to track outcomes of the innovation and provide feedback to the staff related to the impact of the changed practice.

One of the key contextual barriers to spread involved the large and decentralized workforce in the home care sector. Organizations provided services in very large geographic areas and employed large numbers of professional and non-professional staff, making it very challenging to ensure that all staff were well trained and supported in using innovations. The part-time nature of the workforce contributed to these challenges.

## Discussion

This study makes an important contribution to the implementation literature on the spread of best practices. First, the study resulted in a theoretical model that provides a useful blueprint for spreading best practices within home care. Findings highlight the critical role played by passionate and committed leaders at multiple levels (e.g., frontline, managers, directors) of the organization in facilitating spread. Further, the model emphasizes the importance of ‘seeing the benefits’ of the innovation and the particular challenges experienced in home care settings in making this possible. Second, the longitudinal nature of the study revealed the intertwining nature of spread and sustainability processes. Third, study findings accentuated the determining role that contextual factors play in the spread process. Each of these contributions is discussed in the following sections.

The theoretical model of spread illustrates the complexity of the spread process, uniquely combining phases of spread, facilitators of spread, and the context of spread, as well as the intertwining of spread and sustainability. Three intricately entwined process characteristics, specifically sequences, cycles, and spirals, are reflected in the model [49]. The model suggests ways to support home care workers and organizations in planning and implementing spread of innovations. This model of spread is both similar to and distinct from two previously published models of spread [19,20]. All three models identify the perceived need for the innovation, leadership, measurement and feedback, and context as key concepts. The spread model developed in this study is unique in that it integrates five phases of spread with facilitators of spread and reflects the intertwining nature of spread and sustainability. Further, this spread model describes the important roles of different leaders (champions, managers, project leads, and steering committee) in spreading best practices in home care settings. Study findings extend the previous very limited literature on spread in home care organizations [32] through the application of a rigorous grounded theory research approach and the resulting theoretical model of spread.

One of the key facilitators of spread in this study, leading with passion and commitment, is consistent with other studies that have found leadership to be critically important for implementing and sustaining innovations [37-39,51]. This finding is consistent with studies inside and outside of health care settings that have shown that the commitment and attitude of leaders has a significant association with the acceptance and use of new innovations [52,53]. Leadership was the only significant predictor of sustaining practice guideline use 2 and 3 years after implementation in 37 Canadian health care organizations, accounting for 47% of the variance (*p* < .001) [41]. Other studies have found that passionate and persistent leaders and champions used multidimensional, tailored strategies to support adoption of best practices [37,39]. The current study explains how different leaders, specifically managers, champions, and project leads, worked with passion and commitment to spread innovations in home care. This facilitator was largely absent in the non-spread organization.

Another key facilitator of spread found in this study, seeing the benefit, is consistent with findings from other studies of guideline implementation and spread [19,37]. Home care settings experienced particular challenges in obtaining and sharing information on both how broadly the innovation had spread and client outcomes related to spreading the innovation. These challenges were related to factors such as the lack of electronic health records and communication systems. The literature indicates that there are still important knowledge gaps related to when, why, and how audit and feedback interventions are optimally used [54].

Study findings also make an important contribution to our understanding of the intertwining nature of spread and sustainability processes in home care. Data collected at the second interviews demonstrated that following the internal spread phase, resources such as release time for the project lead and for the staff to attend educational sessions related to the innovation were greatly scaled back, putting sustainability in jeopardy. Sustainability of the innovation was not considered as a part of the planning for spread process. Literature suggests that some of the facilitators of spread found in this study may be similar to those that facilitate sustainability of innovations [41,55-58].

Finally, this study makes a valuable contribution to our understanding of how contextual factors at multiple levels influenced the spread process in home care settings. Research has shown that health care reforms to shift the focus of care from costly acute care institutions to home care settings have resulted in heavier workloads, job insecurity, job stress, and decreased job satisfaction for home care workers [59]. The home care workforce is geographically dispersed with many part-time employees, presenting obvious challenges to the spread of new practices. Data from the current study affirm the challenges of introducing and spreading new innovations given heavy workloads and job stress of home care workers [32]. Despite these challenges, three of the four organizations were able to effectively spread best practices by using facilitating strategies to address the organizational and system barriers. In one organization, momentum continues to build as they contribute their tools, processes, and experiences to the development of a province-wide initiative to spread wound care best practices.

Study findings reflect all three forms of data analysis described by Strauss and Corbin [44] as building on one another: description, conceptual ordering, and theorizing. The in-depth descriptions of organizational spread processes (Table 3) reflect a descriptive analysis of spread as it actually happened in these settings. The model that includes phases of the spread process reflects conceptual ordering of events along a temporal dimension. Finally, the facilitators of spread reflect an explanatory scheme (or theory) of factors that determined successful spread of innovations within home care organizations and can be used to guide actions.

### Considerations for practice, policy, and research

There are a number of considerations for practice, policy, and research arising from the study findings. In relation to practice considerations, almost all participants spoke about the value of implementing the innovation on a small scale before moving it to the rest of the organization. They described the value of ensuring it was feasible and applicable and that frontline staff had opportunities to try it out, give feedback, and build buy-in. Another practice implication involves explicitly planning for both spread and sustainability at the start of the change process. It is clear that we cannot afford to invest time and resources in spreading innovations that are not sustained or evaluated and revised over time. While commitment and passion were consistent behaviors among leaders spreading best practices, evidence shows that leadership can be developed and acquired and is not an innate trait [60,61]. Research has found that leadership training can result in significant improvements in knowledge and skill [60]. Thus, it is important to engage leadership teams in setting priorities and directions for implementing, sustaining, and spreading change.

Study findings call attention to policy considerations for spread in home care settings, in particular strategies that address the contextual barriers to spread. Increased investment in the home care sector is key, given the increasing number of older home care clients, limited resources, and escalating costs associated with increased client acuity [22,23]. Investment is necessary to ensure success in the spread of best practices, given study findings of the importance of release time for project leads, educational, and communication activities and high turnover rates.

Findings also highlight the need to ensure the necessary resources for stakeholders to ‘see the benefits’ of spread. Efficient audit and feedback mechanisms allow providers to see the immediate and beneficial effects of practice change on client outcomes. Home care organizations and funders need to invest in electronic health records and feedback systems that facilitate communication both within and across home care agencies.

Further research is needed to test this spread model in other home care settings to confirm its applicability. Inclusion of additional innovations, home care settings, and interviews over a longer term might result in revisions to the model. Research is also needed to explore how external spread happens in and beyond the home care sector, as external spread was not evident in this study. Further research is required to explore the intertwining nature of sustainability and spread and to identify approaches that will ensure both spread and sustainability of effective innovations. Study findings indicate that there was a significant investment of resources in the spread process (e.g., release time for project leads, champions, managers, and steering committees; educational time). However, we do not know the impact of spread on the costs of programs or what factors affect costs of spreading innovations [62]. This is a key research priority given the funding challenges in home care and other health care sectors. Client perspectives would be most valuable to include in future research in particular related to seeing the benefits of the spread of guidelines. Finally, it would also be useful to explore how other theories, such as the normalization process theory that explains the processes by which complex interventions are routinely embedded in health care practice, can potentially complement this theory of spread [63,64].

## Conclusion

The theoretical model provides a new conceptual rendering of the process of the spread of best practices within home care settings. This understanding of the spread process has the potential to facilitate more effective and efficient spread of best practices to improve outcomes for frail older adults, the highest users of home care services. The key concepts of the theoretical model provide a useful guide to inform spread of innovations and may be relevant beyond home care settings.

## Availability of supporting data

Some of the qualitative data set supporting the results of this article are included within the article. A longer report with more details is available from the lead author.

## Definition of spread

We defined spread (or success) as “the process through which new working methods developed in one setting are adopted, perhaps with appropriate modifications, in other organizational contexts” [4]. We considered spread to have occurred if the tools that were implemented on a small scale in a few sites or branches of an organization were then moved to and adopted (perhaps with revisions) in additional organizational sites. This was assessed through constant comparative analysis of all interviews conducted in each organization.

## Abbreviations

BPGs: best practice guidelines
BPSO®: Best Practice Spotlight Organization
CCAC: Community Care Access Centre
RNAO: Registered Nurses’ Association of Ontario
